# Fungal-Bacterial Interactions in the Human Gut of Healthy Individuals

**DOI:** 10.3390/jof9020139

**Published:** 2023-01-19

**Authors:** Evy Maas, John Penders, Koen Venema

**Affiliations:** 1 Centre for Healthy Eating & Food Innovation, Campus Venlo, Maastricht University, Villafloraweg 1, 5928 SZ Venlo, The Netherlands; 2 Euregional Microbiome Center, P. Debyelaan 25, 6229 HX Maastricht, The Netherlands; 3 Department of Medical Microbiology, School of Nutrition and Translational Research in Metabolism (NUTRIM) and Care and Public Health Research Institute (Caphri), Maastricht University, P. Debyelaan 25, 6229 HX Maastricht, The Netherlands

**Keywords:** fungi, microbiome, ITS2 sequencing, fungal-bacterial interactions

## Abstract

Most studies of the microbiota in the human gut focus on the bacterial part, but increasing information shows that intestinal fungi are also important for maintaining health. This can be either by directly influencing the host or by indirectly influencing the gut bacteria that link to host health. Studies of fungal communities in large cohorts are scarce; therefore, this study aims at obtaining more insight into the mycobiome of healthy individuals and how this mycobiome interacts with the bacterial component of the microbiome. For this purpose, ITS2 and 16S rRNA gene amplicon sequencing was performed on fecal samples from 163 individuals which were available from two separate studies to analyze the fungal and bacterial microbiome, respectively, as well as the cross-kingdom interactions. The results showed a much lower fungal, as compared to bacterial, diversity. *Ascomycota* and *Basidiomycota* were the dominant fungal phyla across all the samples, but levels varied enormously between individuals. The ten most abundant fungal genera were *Saccharomyces, Candida, Dipodascus, Aureobasidium, Penicillium, Hanseniaspora, Agaricus, Debaryomyces, Aspergillus*, and *Pichia,* and here also extensive inter-individual variation was observed. Correlations were made between bacteria and fungi, and only positive correlations were observed. One of the correlations was between *Malassezia restricta* and the genus *Bacteroides*, which have both been previously described as alleviated in IBD. Most of the other correlations found were with fungi that are not known as gut colonizers but originate from food and the environment. To further investigate the importance of the observed correlations found, more research is needed to discriminate between gut colonizers and transient species.

## 1. Introduction

The human gut is a complex ecosystem that consists of numerous microorganisms, not only bacteria but also archaea, viruses, and fungi [1,2,3]. Studies concerning human gut microbiota usually focus on the bacterial component of the microbiota, but awareness about the importance of the other components is rising; in particular, the fungal component of the microbiota, the so-called mycobiota, is getting more attention. Among other reasons, the low density of fungi in the gut compared to bacteria has led to the former lack of attention to mycobiota. Fungi that are cultivable are present in the range of 10^2^ to 10^6^ CFU/g [4], compared to 10^11^–10^12^ CFU/g for bacteria [5]. The fungal genes take up around 0.1% of the total microbial metagenome [6]. In addition, the more complex molecular and phylogenetic characterization of fungi has contributed to the neglect of this part of the microbiome community. Historically, fungi were difficult to study with the use of culture-dependent techniques, as many fungi are difficult to culture outside the body, leading to an incomplete picture of gut fungal composition [7]. With the rise of culture-independent techniques, increasing information was also gathered on the mycobiome. In bacteria, the use of 16S rRNA gene amplicon sequencing is standard practice, but different regions of ribosomal RNA have been used for studying the mycobiome. Although 18S rRNA sequencing is often used, it is known that 18S primers amplify non-fungal species, e.g., contamination from host cells or food [8,9]. In addition, the identification is limited to the family or genus level by the use of 18S rRNA sequencing. Therefore, in recent years the regions that are preferred for the sequencing of fungi are the ITS regions. The ITS units are the spacer DNA regions between the small-subunit (SSU or 18S) rRNA and the large-subunit (LSU or 28S) rRNA genes, where ITS1 is positioned between the 18S and 5.8S rRNA genes and ITS2 between the 5.8S and 28S genes. When using the ITS regions for the identification of fungi, the whole ITS region can be used, but the separate use of the ITS1 and ITS2 is described [1,9,10].

These new insights obtained through next-generation sequencing of the mycobiome show that fungi are important in several gut-associated and metabolic diseases [11,12,13]. The gut mycobiome could also be a reservoir for opportunistic pathogens, which can grow out and cause infections when the gut ecosystem is disturbed [14,15]. In addition to linking fungi to disease states, it is also essential to define the mycobiome composition in a healthy state and to determine how the mycobiome is intertwined with the bacterial component of the microbiome. These fungal–bacterial cross-kingdom interactions could give more insights about the complex ecosystem that exists in the gut.

Studies of the gut mycobiome and microbiome in healthy individuals in a larger cohort are very limited. Therefore, this study aimed to analyze the gut fungal composition and discover fungal–bacterial interactions in 163 healthy individuals.

## 2. Materials and Methods

### 2.1. Study Subjects

The volunteers for this study were healthy individuals who participated in two prior studies; in one of these, the microbiota was analyzed to determine what could be the core microbiota in healthy individuals, and in the other the difference in the gut microbiota of healthy individuals and participants with acne was studied (both studies are unpublished). These previous studies did not require medical ethical approval for the collection of fecal samples. All the donors provided informed consent. In total, 163 persons were asked to provide fecal samples. From these persons, information on age, sex, weight, length, and medication use was collected three months prior to the collection of the fecal samples. The subjects gave written consent for the determination of the microbial composition.

### 2.2. Sample Collection

The volunteers were provided with a fecal collection kit for the collection of the fecal samples. The kit consisted of a fecal collection tube with a scoop containing 9 mL DNA shield (Zymo Research, Irvine, CA, USA) and a fecal collection paper (Fe-Col^®^). The volunteers were asked to donate one fecal sample according to the instructions provided with the kit. The DNA shield buffer stabilized the nucleic acids, which allowed for transporting and storing the samples at ambient temperatures for a longer time [16]. The collection kit was sent to the laboratory facility, where upon arrival the samples were kept at 4 °C until further processing.

### 2.3. DNA Isolation

To determine the fungal and bacterial composition of the fecal samples, the DNA was isolated using the QIAamp Fast DNA Stool Mini Kit (Qiagen, Venlo, The Netherlands). Fungi have a stronger cell wall containing chitin; therefore, a bead-beating step was introduced in addition to the protocol from the kit as adapted from [17]. In short, 500 µL of fecal sample was transferred to a Precellys tube (Bertin Corp, Rockville, MD, USA) which contained 0.5 mm glass beads. Then 1 mL InhibitEX buffer (Qiagen) was added, and the samples were treated in the Precellys 24 homogenizer (Bertin Corp) at 6000RPM for 3 × 30 s. The samples were cooled on ice in between sessions. Next, the samples were heated to 95 °C for 7 min, subsequently mixed by vortexing, and the sample was pelleted by centrifugation for 1 min at 14,000× *g*. Thereafter, 30 µL proteinase K was added, and the steps were followed as described in the QIAamp Fast DNA Stool Mini Kit handbook. The final elution step was performed in the smaller volume of elution buffer (50 µL) to increase the DNA yield. After elution, the DNA concentration was determined using the Qubit 1X dsDNA high sensitivity (HS) assay and a Qubit 3.0 Fluorometer (Invitrogen, Waltham, MA, USA).

### 2.4. Bacterial Composition

Isolated DNA was used for the determination of the bacterial composition. Previously, the V3-V4 region of the 16S rRNA gene had been sequenced. The Illumina protocols for 16S rRNA metagenomic sequencing were used for the library preparation (Nextera XT DNA Library Preparation Kit, Illumina, Eindhoven, the Netherlands). For the first PCR, the following primers were used: 341F (5′-CCTACGGGNGGCWGCAG-3′) and 785R (5′-GACTACHVGGGTATCTAATCC-3′) [18]. In the second PCR, Illumina indexes and adapters were added to the sequences (Illumina Nextera XT Index Kit v2 Set A). After the library preparation, the DNA was quantified using the Qubit 1X dsDNA HS assay and a Qubit 3.0 Fluorometer. The length and quality of the sequences were checked on the Bioanalyzer (Agilent, Santa Clara, CA, USA) using the DNA 1000 kit. Subsequently, the samples were pooled in an equimolar manner and loaded on the reagent cartridge; then sequencing was performed on the Miseq system (Illumina Miseq reagent kit v3). Fastq files were generated with the use of the Local Run Manager Generate FastQ module v3.

### 2.5. Fungal Composition

In addition to the bacterial composition, the isolated DNA was also used for the determination of the fungal composition. For this purpose, the internal transcribed spacer unit 2 (ITS2), as the phylogenetic marker for the identification of fungi [19], was sequenced. ITS2 was chosen because it is more conserved than ITS1 [20]. The Illumina “Fungal Metagenomic Sequencing Demonstrated Protocol” was used for the library preparation (Nextera XT DNA Library Preparation Kit, Illumina). In the first PCR, the primer pair ITS F (5′-GCATCGATGAAGAACGCAGC-3′) and ITS R (5′-TCCTCCGCTTATTGATATGC-3′) was used [21]. Illumina indexes and adapters were added in the second PCR (Nextera XT Index Kit v2 Set A, Illumina). After the library preparation, the DNA was quantified (Qubit 1X dsDNA HS assay), and the quality and length of the sequences were analyzed using the Bioanalyzer in combination with the DNA 1000 kit (Agilent). Because of the variable lengths of the sequences from the ITS2 regions, all the fragment sizes were checked and the average length was determined to calculate the appropriate concentration for equimolar pooling in the subsequent steps. The library was sequenced on the Miseq system using the Illumina reagent kit v3. Fastq files were generated as described above.

### 2.6. Bioinformatics Analysis

Fastq files for both bacteria and fungi generated with the Miseq system were analyzed with the Quantitative Insights Into Microbial Ecology 2 (QIIME2) software package (version 2019.7) [22,23]. To optimize the quality of the ITS2 fastq files, the QIIME2 plugin Q2-ITSxpress plugin was used to trim the ITS sequences [24]. The dada2 plugin was used for both bacteria and fungi libraries for demultiplexing, quality filtering, and denoising [25]. Taxonomic analysis was performed with the q2-feature-classifier [26], using the SILVA database (version 132) for bacterial identification and the UNITE database (version 02-02-19) for fungal identification.

### 2.7. Statistics

Alpha-diversity indexes (observed features and effective Shannon) were determined using QIIME2. The data was visualized using GraphPad Prism 9.3.0. Differences between groups were tested with the Kruskal-Wallis test, with the Dunn’s test as post hoc analysis when significant differences (*p* < 0.05) were found. These analyses were done in Rstudio using the packages ggpubr and FSA. Further analysis was performed in Rstudio using the packages qiime2R, phyloseq, ggplot2, and ComplexHeatmap. All the analyses were performed using R version 4.04.

## 3. Results

To study the gut mycobiota and microbiota in healthy individuals, ITS2 and 16S rRNA amplicon-based sequencing was performed on the fecal samples of 163 individuals. The study population consisted of 38 (23.3%) male and 125 (76.7%) female subjects, with an average age of 35.5 years and an average BMI of 22.5 (Table 1).

**Figure 1:**
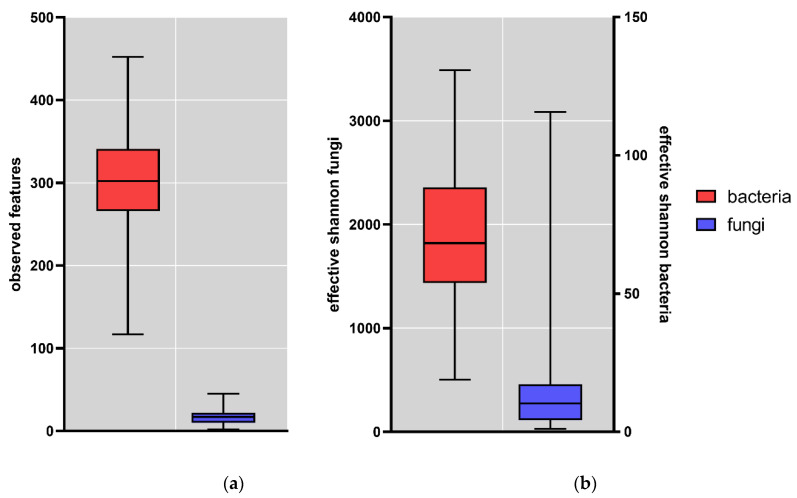
Box plots of alpha-diversity indexes of the fecal samples (N = 163); (**a**) Observed features fungi (blue) and bacteria (red); (**b**) Effective Shannon fungi (blue) and bacteria (red). Whisker: min to max; horizontal line = median; box = 25th to 75th percentile.

In the 163 samples, the “observed OTUs” diversity index was determined on ASV level. The observed ASVs were lower for fungi compared to bacteria (Figure 1a). In the fecal samples, on average 16.9 (range 2–45) fungal and 298.4 (range 117–452) bacterial ASVs were observed. To further assess the alpha diversity, the effective Shannon diversity index was determined (Figure 1b); this was also lower for fungi (17.29; range 1.07–115.67) compared to bacteria (1475; range 503.03–3487.31). These results show that the gut fungal population is far less diverse than the bacterial population.

The lower diversity for fungi compared to bacteria here is comparable to what has been observed in previous studies. In the study on the mycobiome of the Human Microbiome Project healthy cohort, the metric “observed OTUs” ranged from 9 to 92 with a mean of 14 OTUs [27], in close agreement with the value observed in the current study. In addition, Shuai et al. observed lower alpha-diversity values for fungi compared to bacteria [28].

**Figure 2:**
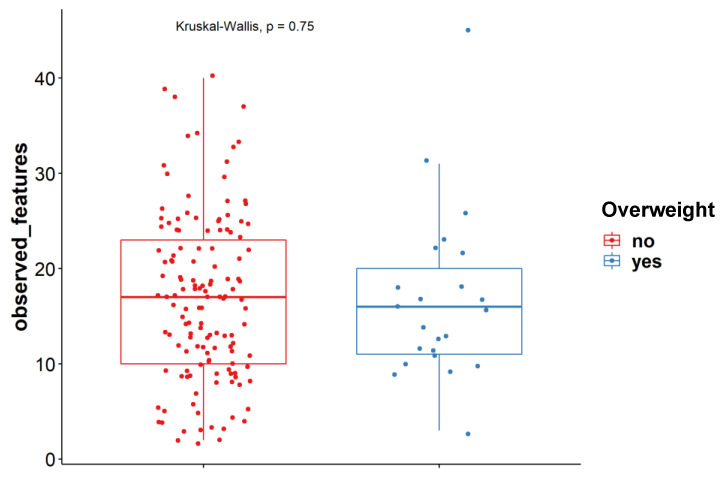
Observed features at ASV levels for overweight (n = 23) and non-overweight (n = 140) subjects (Kruskal-Wallis test).

We did not observe an association between a higher BMI (>25) and the alpha-diversity indexes either for bacteria or for fungi (Figure 2). This is in contrast to what was described in earlier studies, where decreased fungal diversity was observed in overweight as compared to lean subjects [29]. This discrepancy might be explained by the fact that the subjects in this study were not selected to create two even groups with overweight and non-overweight persons as was the case in the study by Mar Rodríguez et al. In our study, N = 23 for overweight individuals (Table 1) and N = 2 for obese individuals probably does not lead to the proper statistical power. In addition, there was no sex difference in the mycobiome (Figure S1). Moreover, no differences were observed in individuals of different ages (Figure S2).

**Figure 3:**
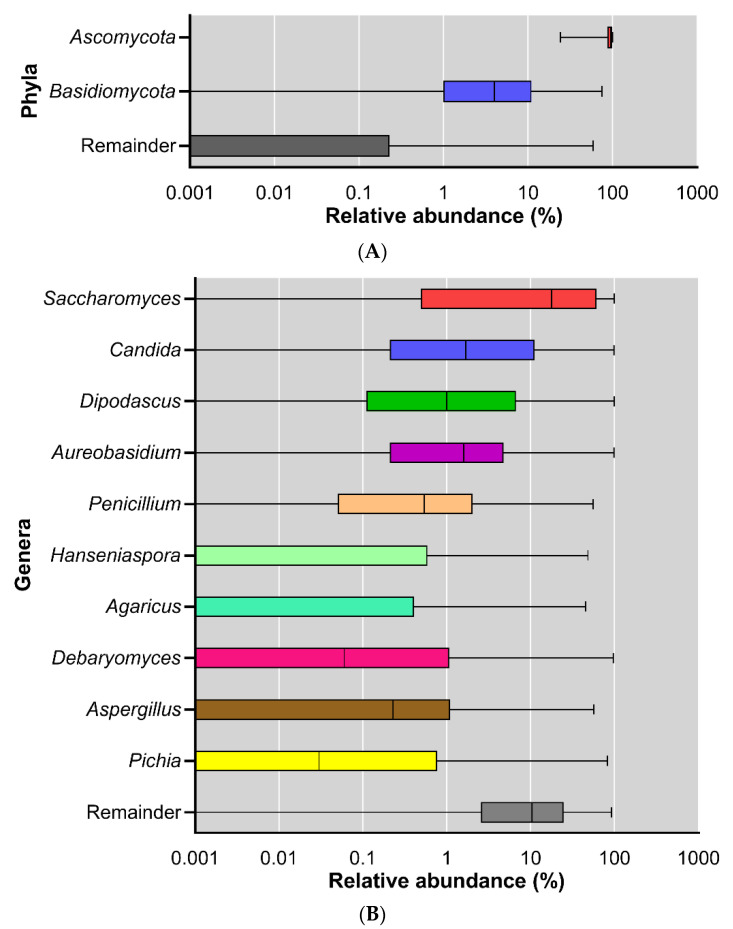
Relative abundance of fungi across all subjects; displayed at log scale; (**A**) phylum level; (**B**) genus level.

In addition to the diversity analyses, the fungal samples were also studied for their taxonomic composition. At the phylum level, *Ascomycota* and *Basidiomycota* were the dominant phyla across all the samples, where the average relative abundance was 89.9% ± 14.4% (mean ± SD) for *Ascomycota* and 9.30% ± 14.4% (mean ± SD) for *Basidiomycota*. However, the inter-individual variation in the relative abundance of these dominant phyla was extremely large (Figure 3A). The dominance of *Ascomycota* and *Basidiomycota* corresponds with earlier findings in other mycobiome studies [27,28]. Mar Rodríguez et al. described the presence of *Ascomycota, Basidiomycota*, and *Zygomycota* in fecal samples [29].

The mycobiota composition was also analyzed at the genus level. The ten most abundant genera were: Saccharomyces, Candida, Dipodascus, Aureobasidium, Penicillium, Hanseniaspora, Agaricus, Debaryomyces, Aspergillus, and Pichia (Figure 3B).

Many of the genera that dominated in our study (Figure 3B) also belonged to the dominant genera in previous studies on the mycobiome (Table 2). This suggests that some fungi persistently colonize our gut and are not just transient passengers of the gastrointestinal tract when ingested with the diet.

However, from our data and from the literature it can be observed that there is a high inter-individual variability in the mycobiome, and when compared to bacteria, fungi are less stable over time [30]. Some of this high variability can still be explained by those fungi that are transiently introduced in the human GI tract via diet or environment, despite the fact that most genera seem to have been observed in other studies before and therefore are likely gut colonizers. It has been described that fungi and fungal DNA can be found in the diet of humans [31,32,33]. Some of these fungal genera are not able to grow under the conditions found in the gut (temperature, pH, and low oxygen levels) [34]. An attempt to discriminate between fungi found in the diet and in fecal samples was made using mouse models, where fungi found in chow were compared with fungi in feces. Some genera were found both in the chow and in the feces, and others were only found in the feces and not in the chow [35,36]. The recent study performed by Shuai et al. described the possibility of a core mycobiota that is more stable, because from their data there were some core fungal taxa that were found in samples of individuals on two time points 3 years apart [28]. Since in our study we only had one time point at which the fecal samples were collected, it is difficult to discriminate between the transient species that originate from the diet and true colonizers that are consistently found in the gut.

**Figure 4:**
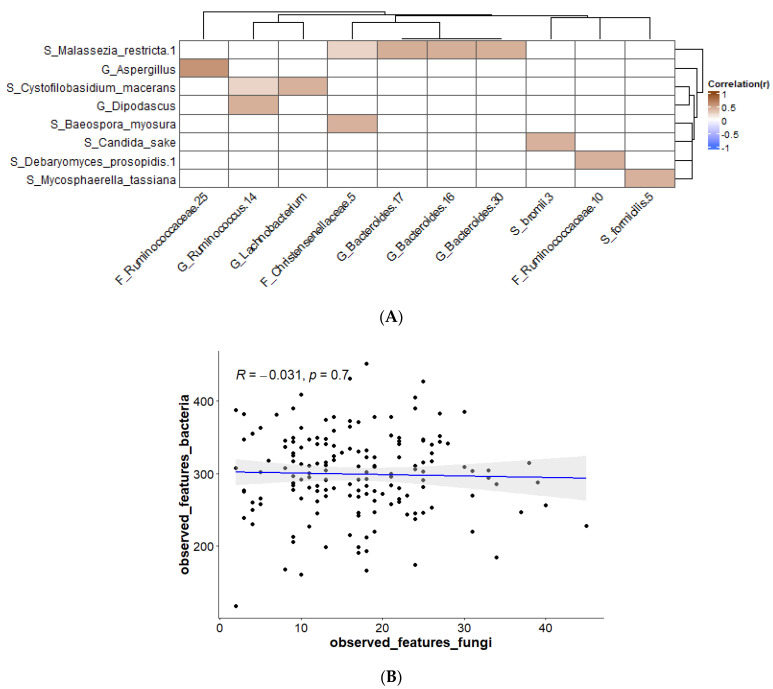
(**A**) Bacterial–fungal correlations using Spearman correlation present in ≥10% of samples; corrected p-value after FDR cut-off 0.1; (**B**) Spearman correlations for observed features at ASV levels for bacteria versus fungi.

To give more insight on how the mycobiome is shaped in healthy individuals, interactions with the bacterial component of the microbiome were investigated. This was done by correlation of fungal ASVs with bacterial ASVs (by Spearman correlation with FDR correction). In the supplemental material, correlations at genus level can be found (Figure S3). Figure 4A shows correlations that are present in at least 10% of the study subjects. The picture shows that only positive correlations were found. There are several examples of positive correlations found in the literature. For example, the secretion of amino acids by the yeast *Saccharomyces cerevisiae* promotes the bacteria *Lactiplantibacillus plantarum* and *Lactobacillus delbrueckii* subsp. *lactis*, which in turn produce lactose that can be used by *S. cerevisiae* [37]. In addition, it was found that *Candida* spp. could aid in the growth of *Clostridioides difficile* when grown anaerobically [38]. Bacteria from the family *Enterobacteriaceae* were shown to promote the colonization of *Saccharomyces boulardii* or *Candida albicans* in mouse gut [39]. There are some suggestions of how these positive relationships work, e.g., several studies show that fungi and bacteria are involved in combined biofilms, which creates a favorable environment for both the bacteria and the fungi [40,41]. Another mechanism is quorum sensing, where molecules produced by members of one kingdom can promote the growth of members of the other kingdom [42]. Furthermore, fungi can create a strict anaerobic environment by the consumption of oxygen via the mitochondria, which can be beneficial for certain strict anaerobic bacteria [41]. In our study, however, fungal–bacterial correlations were found that have not been described in the literature before (Figure 4). Although the number of fungal and bacterial species found in the gut is high, the information on fungal–bacterial interactions is scarce. For part of these sorts of experiments, the fungi and bacteria that are investigated are researched in vitro, outside of the complex environment in which they normally live; because of the available research, which is mostly limited to in vitro studies, some interesting relationships may have been missed [43].

Correlations between the different taxa were studied. Two areas of correlation were observed in the data: for bacterial taxa between themselves (Figure S4; upper left quadrant) and for fungal taxa between themselves (Figure S4; lower right quadrant). There were only a limited number of correlations between bacteria and fungi (Figure 4A), likely due to the high inter-individual variation in taxa, particularly for fungi. Figure 4A shows that in our study *Malassezia restricta* is positively correlated with the genus *Bacteroides*. *M. restricta* has been described as a member of the skin microbiota, but it is also seen in other studies on the gut mycobiome, so the presence of this fungus in the fecal samples is probably through introduction via skin contact [44,45]. In patients with Crohn’s disease, the presence of *M. restricta* in the colonic mucosa is observed, and in mice *M. restricta* can induce colitis [46]. In addition, *Bacteriodes* spp. have shown to play a role in inflammatory bowel disease (IBD) [47,48]. These examples show that both fungi and bacteria are disturbed in disease and that interactions possibly have a role in this. *Candida sake* is a yeast that has been isolated from sake; it is found as a spoilage organism in a range of food products such as grape juice, sauerkraut, and frozen salmon [49]. *C. sake*, which is correlated to *Ruminococcus bromii*, does not grow at 37 °C, so it should be a transient species. *R. bromii* is a species described to be important in starch degradation [50]. Cross-feeding of bacteria with glucose formed from starch that has been degraded by *R. bromii* has been described [51,52]. It is possible that such interactions also take place between fungi and bacteria. *Mycosphaerella tassiana* has been described as a fungus present in the airway mycobiota and which could possibly have entered the gut via this route [53,54]. The link with *Gemmiger formicilis* (Figure 4) is not described in the literature. *Aspergillus* spp. are commonly found in soil, air, or plants, but they can survive at 37 °C. Their presence in the gut is most likely from environmental origin [55,56]. *Aspergillus* has been described to be involved in lung diseases, where it can cause dangerous clinical infections [57]. Aspergillosis can also affect the gut [58]. In our study, *Aspergillus* is positively correlated with *Ruminococcaceae,* which is different than has been described in the piglet gut, where a negative correlation was found [59]. *Dipodascus* was shown to be one of the most abundant genera associated with people living in a rural area [60]. Moreover, *Ruminococcus* spp. are found in the gut of inhabitants of a rural area, and they play a role as important plant degraders [61]. The other fungi found in this study, *Cystofilobasidium macerans, Baeospora myosura*, and *Debaryomyces prosopidis,* which were shown to have interactions with bacteria, have not been well described in relation to the human body. They originate from the environment and are probably transferred to the gut via the airway or food; most likely they are transient species and not true gut colonizers [62,63,64]. The analysis of a single fecal sample for each individual does not allow one to discriminate between species that are passing through the GI tract and true gut colonizers. More research is needed in order to discover if these interactions can be relevant to gut health, for example with longitudinal data from subjects with more information on dietary intake. There was no correlation between observed features of fungi and observed features of bacteria (Figure 4B), adding to the large inter-individual variation that was observed between the participants.

## 4. Conclusions

The aim of this study was to describe the mycobiota of a large cohort of healthy individuals and to see if fungal–bacterial cross-kingdom correlations could be observed. Comparable to other studies, the mycobiome in this study was shown to have low diversity and large variation between individuals. Diversity indexes were compared for different BMI—overweight vs not overweight—but no significant differences were observed. This contrasts with other studies but could possibly be explained by the fact that this study population was not selected to equally represent overweight and non-overweight subjects. Taxonomic classification showed dominance of the phyla *Ascomycota* and *Basidiomycota*, but the ratio varies greatly between individuals. This dominance is similar to what was described in other studies; this is also the case for the majority of the most-abundant genera that were found, such as *Candida, Saccharomyces, Penicillium*, and *Aspergillus.* From the taxonomic classification, large inter-individual differences could also be observed, possibly explained by the fact that many fungi are introduced via the diet or environment and do not stably colonize the gut. However, a few genera were observed by us and in other studies, possibly representing a core mycobiota. Due to the single sample point in time, the nature of this study did not allow us to discriminate between transient species and gut colonizers. This was also found when looking at fungal–bacterial interactions, where most of the interactions found were with fungi that were hypothesized to be derived from the environment. All the interactions found were positive correlations. These kinds of correlations have been described before in the literature, but the relations observed in this study were new. More research is needed to investigate the relevance of these interactions.

## Figures and Tables

**Table 1:** Characteristics of study subjects (N = 163).

Sex	38 male (23.3%); 125 female (76.7%)
Age ^a^	35.5 ± 12.1 years
Weight ^a^	67.9 ± 11.3 kg
Length ^a^	173.5 ± 8.7 cm
BMI ^a^	22.5 ± 2.7
Overweight ^b^	23

^a^ = mean ± SD; ^b^ BMI > 25.

**Table 2:** Most abundant genera in mycobiome studies. In bold overlap with genera found in this study.

Most Abundant Genera	Reference
***Penicillum*** *, **Candida**, **Saccharomyces**, Mucor, **Aspergillus***	[29]
***Saccharomyces, Candida*** *, **Aspergillus**, Malassezia*	[28]
***Saccharomyces*** *, Malassezia, **Candida**, Cyberlindnera, **Penicillium**, Cladosporium, **Aspergillus**, **Agaricus**, Fusarium, **Pichia**, Debaryomyces, Galactomyces, Alternaria, Clavispora*	[27]

## Data Availability

The raw sequences and corresponding metadata will be archived in the Sequence Read Archive (SRA) repository at the NCBI upon acceptance of the manuscript: http://www.ncbi.nlm.nih.gov/bioproject/908110.

## Supplementary Materials

The following supporting information can be downloaded at: https://www.mdpi.com/article/10.3390/jof9020139/s1; Figure S1. Observed features at ASV levels for female (n=125) and male (n=38) subjects (Wilcoxon test); Figure S2. Spearman correlations for observed features at ASV levels for different ages; Figure S3: Correlations between bacterial and fungal genera present in ≥2% samples; corrected p-value after FDR cut-off 0.2; Figure S4. Spearman correlations for all taxa (bacterial and fungal) present in at least 20% of the samples. The horizontal and vertical lines indicate the division between the kingdoms.
