# Supplementary material for: Fungal-Bacterial Interactions in the Human Gut of Healthy Individuals
Source: J Fungi (Basel). 2023 Jan 19;9(2):139. doi: 10.3390/jof9020139 (PMC9965947; doi:10.3390/jof9020139)
Supplement: Supplementary file 1 [file jof-09-00139-s001.zip › jof-2109184-supplementary.pdf]

Supplemental material

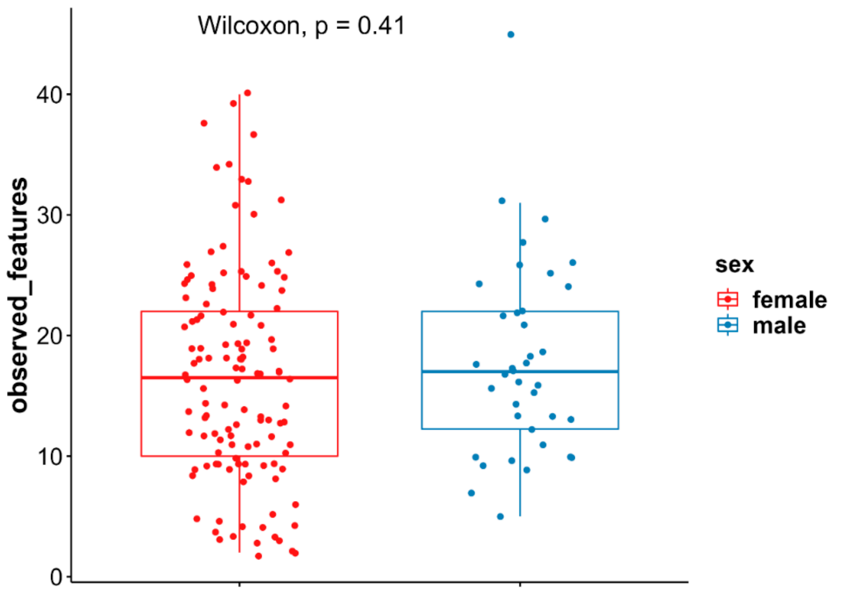

Figure S1. Observed features at ASV levels for female (n=125) and male (n=38) subjects (Wilcoxon test).

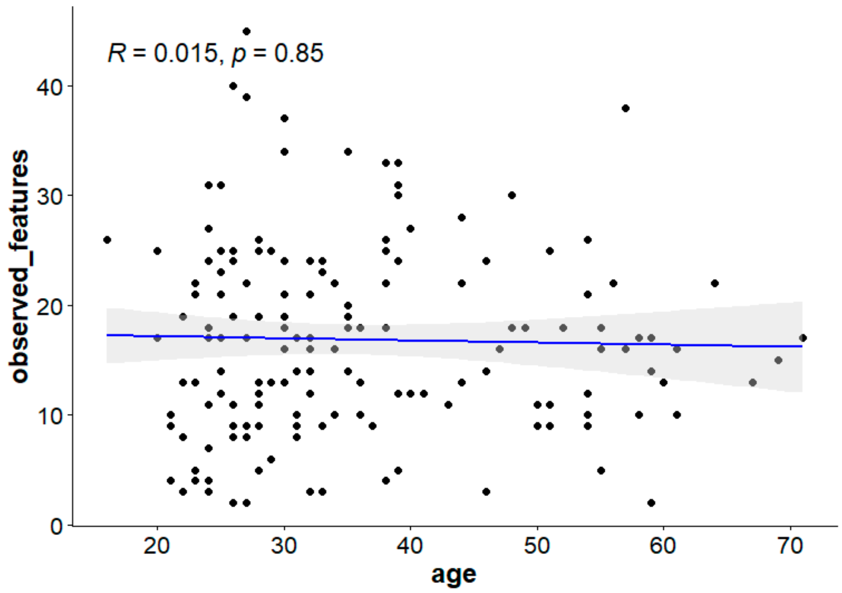

Figure S2. Spearman correlations for observed features at ASV levels for different ages.

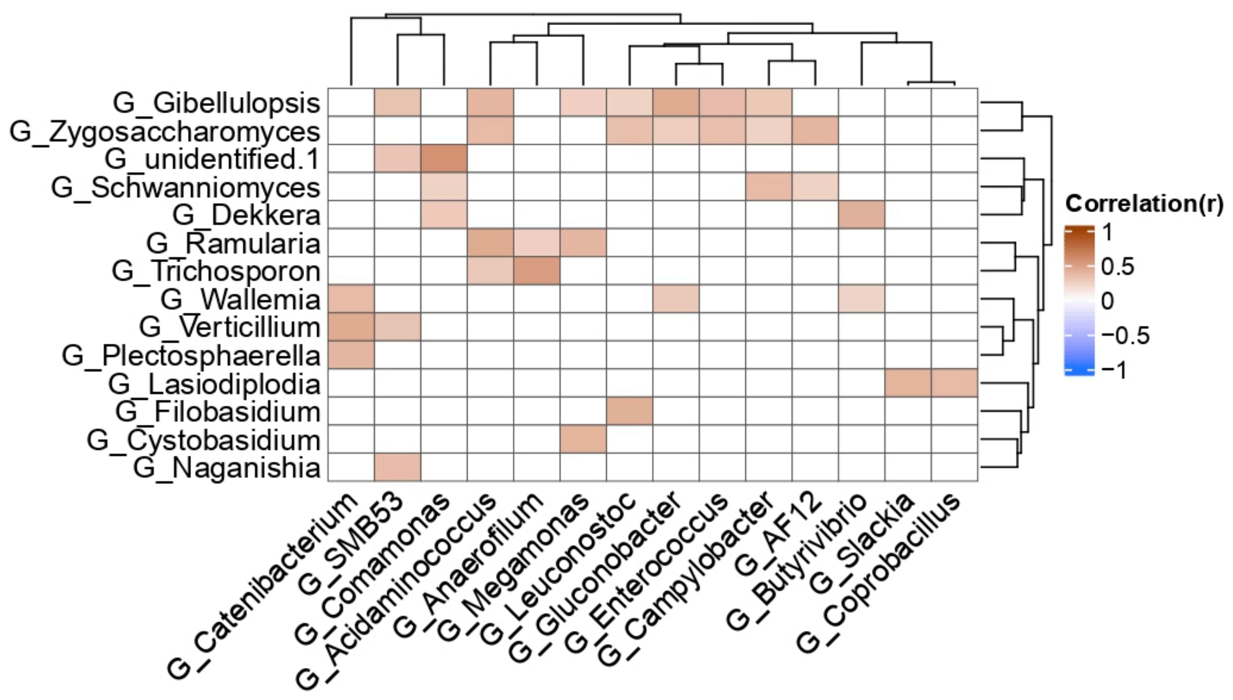

Figure S3. Correlations between bacterial and fungal genera present in ≥2% samples; corrected p-value after FDR cut-off 0.2.

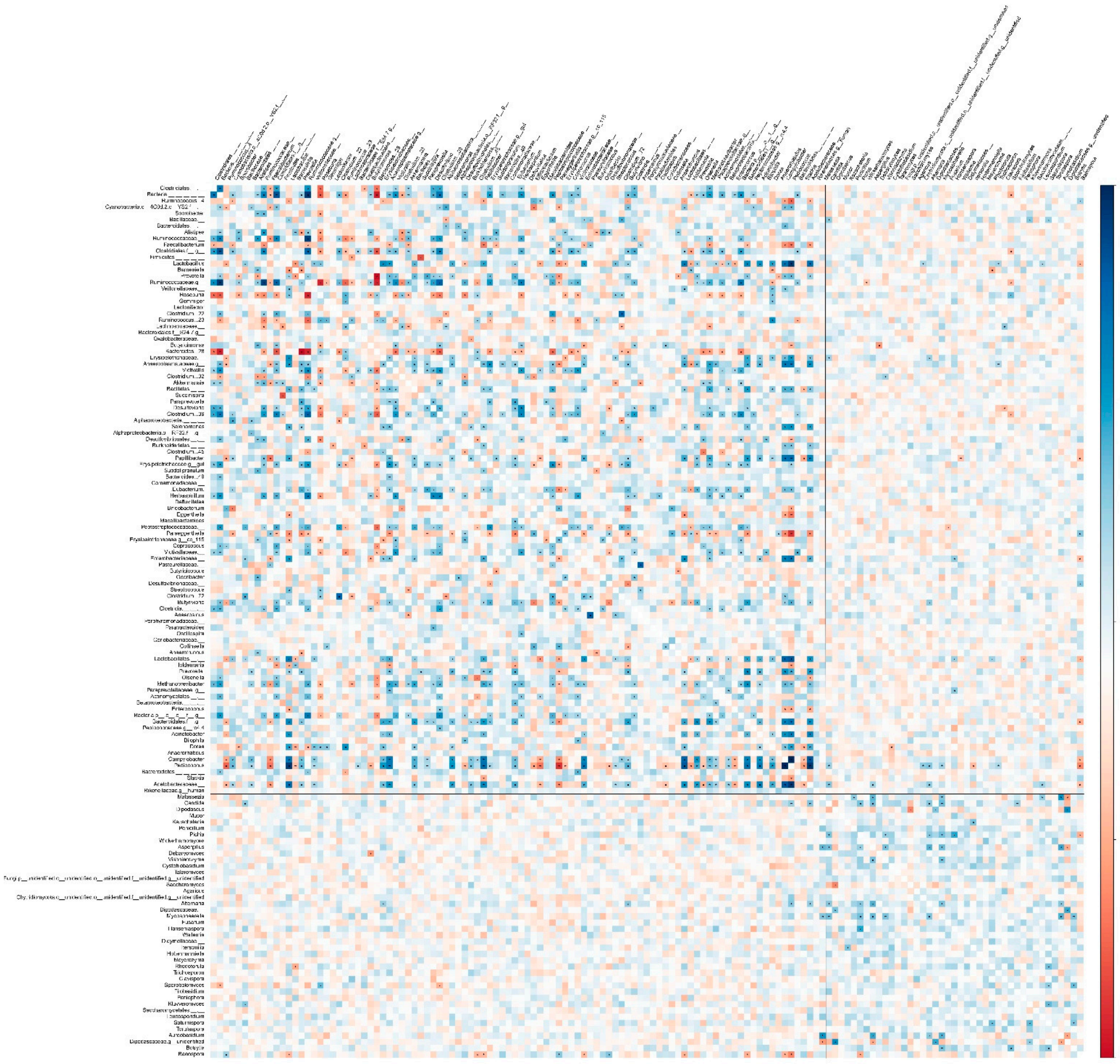

Figure S4. Spearman correlations for all taxa (bacterial and fungal) present in at least 20% of the samples. The horizontal and vertical lines indicate the division between the kingdoms.
